# Comparative Analysis of Microbial Detection in Traditional Culture Versus Metagenomic Next-Generation Sequencing in Patients with Periprosthetic Joint Infection: A Prospective Observational Study

**DOI:** 10.3390/microorganisms14010233

**Published:** 2026-01-20

**Authors:** Po-Yu Liu, Hung-Jen Tang, Susan Shin-Jung Lee, Chun-Hsing Liao, Chien-Hsien Huang, Han-Yueh Kuo, Wang-Huei Sheng, Taiwan Metagenomic Sequencing Microbiology Study Group

**Affiliations:** 1 Department of Internal Medicine, Taichung Veterans General Hospital, Taichung City 407, Taiwan; liupoyu@gmail.com; 2 Department of Internal Medicine, Chi-Mei Memorial Hospital, Tainan City 71004, Taiwan; 8409d1@gmail.com; 3 Department of Internal Medicine, Kaoshiung Veterans General Hospital, Kaoshiung 813414, Taiwan; ssjlee@gmail.com; 4 Department of Internal Medicine, Far-East Memorial Hospital, New Taipei City 220, Taiwan; liaochunhsing@gmail.com; 5 Department of Internal Medicine, National Taiwan University Hospital Hsin-Chu Branch, Hsin-Chu City 300, Taiwan; jama001689@gmail.com (C.-H.H.); hykuo1289@gmail.com (H.-Y.K.); 6 School of Medicine, National Taiwan University College of Medicine, Taipei City 10051, Taiwan

**Keywords:** metagenomic next-generation sequencing (mNGS), periprosthetic joint infection (PJI), synovial fluid, staphylococci

## Abstract

Identifying pathogens causing periprosthetic joint infection (PJI) is a challenge for clinicians. We aimed to evaluate the application of metagenomic next-generation sequencing (mNGS) to identify pathogens in PJI. A prospective analysis was conducted of patients diagnosed PJI between 2022 and 2024 at twelve hospitals in Taiwan. Both conventional bacterial culture (CMT) and mNGS of joint fluid and debrided tissue were performed. Demographic characteristics, laboratory results and clinical outcomes were collected. The diagnostic performance of these two methods was analyzed. A total of 42 patients with a mean age of 67.9 years were enrolled in analysis. The knee was the most common joint involved (69.1%). A high proportion of patients (78.6%) received prior antibiotics within the two weeks at sample collection. mNGS identified pathogens in 28 out of 42 patients (66.7%), whereas CMT yielded positive results in 12 out of 42 patients (28.6%) (McNemar’s test, *p* = 0.01). *Staphylococcus* species was the most common genus detected (*n* = 11), followed by *Cutibacterium* (*n* = 4). Other detected genera included *Escherichia*, *Mycobacterium*, *Enterobacter*, *Klebsiella* (*n* = 2 each), *Acinetobacter*, and *Corynebacterium* (*n* = 1 each). Our results support the idea that mNGS could serve as a valuable diagnostic tool for PJI in addition to traditional culture methods.

## 1. Introduction

Periprosthetic joint infection (PJI) is a devastating complication following joint replacement surgery, imposing significant burdens on patients, healthcare systems, and society. Patients with PJI experience debilitating pain, reduced joint function, impaired mobility, and a diminished quality of life [1,2]. The infection often necessitates prolonged antibiotic treatment, multiple surgeries, and extended hospital stays, leading to physical, emotional, social, and economic hardship [3]. Moreover, PJI is associated with a concerning mortality rate, reported as high as 4% within 90 days of diagnosis and 24–26% in some long-term studies [4,5]. The economic impact of PJI is equally substantial, with annual hospital costs related to PJI of the hip and knee in the United States projected to reach $1.85 billion by 2030 [6]. This growing financial burden stems from the increasing incidence of PJI, coupled with the high costs associated with surgery, prostheses, antibiotics, and prolonged hospitalization [6].

While the incidence of PJI varies in the literature, it generally ranges from 0.5% to 2% of patients undergoing joint replacement surgery, with rates of 0.39% to 3.9% reported after total knee arthroplasty (TKA) [5,7]. With the rising number of prosthetic joint implantations worldwide, the risk and burden of PJI are expected to escalate [8]. Early and accurate diagnosis of PJI is crucial for successful treatment, yet differentiating between septic and aseptic failures remains a significant challenge, as no “gold standard” diagnostic test exists [9]. Conventional microbiological methods, primarily relying on culture, can be insensitive and time-consuming, frequently resulting in culture-negative results. This diagnostic uncertainty can delay appropriate treatment and contribute to poorer patient outcomes.

Metagenomic next-generation sequencing (mNGS) offers a culture-independent approach to pathogen detection, capable of identifying a broad spectrum of microorganisms directly from clinical samples. This technology has the potential to overcome the limitations of traditional culture-based methods, particularly in cases of slow-growing, fastidious, or difficult-to-culture organisms. In the clinical management of periprosthetic joint infection, pathogens are often present in low abundance or suppressed by prior antimicrobial therapy, leading to a high rate of culture-negative cases. Therefore, such highly sensitive technology is particularly vital in orthopedic settings to identify cryptic infections that would otherwise remain undiagnosed by traditional means [10,11]. In this prospective, multicenter study conducted across seven hospitals in Taiwan, we evaluated the diagnostic utility of mNGS in 42 patients with suspected PJI, diagnosed according to the 2021 EBJIS/ESCMID criteria. We analyzed synovial fluid, pus, tissue, and blood samples using both mNGS and conventional microbiology. By comparing mNGS results with conventional microbiological findings and assessing the impact of mNGS on clinical management through clinician surveys, we aimed to determine the value of this novel technology in improving the diagnosis and treatment of PJI. Our findings demonstrate that mNGS significantly enhances pathogen detection rates, particularly in culture-negative cases, and provides clinically actionable information, highlighting its potential to improve the management of this challenging complication.

## 2. Materials and Methods

### 2.1. Study Population

This study was a multicenter prospective trial involving twelve hospitals in Taiwan (Taiwan Metagenomic Sequencing Microbiology Study Group), including the Chi Mei Medical Center, Chinese Medical University Hospital, Far-Eastern Memorial Hospital, Kaohsiung Chang-Gung Memorial Hospital, Kaohsiung Veterans General Hospital, Mackay Memorial Hospital, National Taiwan University Hospital, National Taiwan University Hospital Hsin-Chu Branch, National Taiwan University Hospital Yun-Lin Branch, Shin-Kong Memorial Hospital, Taichung Veterans General Hospital and Tri-Service General Hospital.

In this prospective study, patients with suspected PJI who underwent surgery at our center between May 2022 and May 2024 were sequentially enrolled. The diagnosis of PJI was based on the European Bone and Joint Infection Society (EBJIS)/ESCMID definition established in January 2021. We recorded demographic characteristics, medical histories, physical examination findings, serum inflammatory indexes, synovial fluid white blood cell count (SF-WBC), conventional microbial test results (cultures from sterile sites or pus), and mNGS findings. Causative pathogens were identified through an integrated adjudication by at least two independent infectious disease specialists, incorporating microbiological findings, sequencing metrics, and clinical context.

### 2.2. Ethics Declaration

This study has been approved by the Institutional Review Boards (Ethics Committee) of National Taiwan University Hospital (IRB No. 202203034RINC) and the Ethics Committee at each participating hospital.

### 2.3. Nucleic Acid Extraction

For genomic DNA extraction, 600 μL of the synovial fluid specimen was transferred into a 2 mL Lysing Matrix tube containing 1 g of 0.5 mm diameter glass beads. The tube was placed on a FastPrep-24 5G instrument (MP Biomedicals, Irvine, CA, USA) at a speed of 10 m/s for a total of 25 min. The supernatant (200 μL) was then transferred to a new 1.5 mL tube after a high-speed centrifugation at 8000× *g* for 1 min for further DNA extraction. Genomic DNA (gDNA) extraction was performed using the TIANMicrobe Pathogen DNA Kit (TIANGEN BIOTECH, Beijing, China) according to the manufacturer’s instructions. The total gDNA was quantified using the Qubit dsDNA HS Assay Kit on a Qubit 4.0 Fluorometer (Thermo Scientific, Waltham, MA, USA) [12].

### 2.4. DNA Library Preparation

Approximately 100 ng of input genomic DNA (gDNA) was used for DNA library construction, following the MGIEasy FS DNA Library Prep Kit protocol (MGI, Shenzhen, China). The procedure included DNA fragmentation, end-repair, adapter ligation, and PCR amplification. In brief, DNA fragmentation was performed using an enzymatic method with a fragmentation enzyme to generate DNA fragments of approximately 150–250 bp. This was achieved by incubating the sample at 32 °C for 20 min. The fragmented products were purified using 0.8× DNA purification beads, followed by two washes with fresh 80% ethanol, and eluted in 42 µL of TE buffer. The purified DNA fragments were end-repaired and A-tailed under the following conditions: 37 °C for 10 min, followed by 65 °C for 15 min. Adapters were then ligated to the end-repaired and A-tailed DNA fragments at 23 °C for 20 min, followed by bead purification at a 0.5× ratio. Each library was assigned a unique barcode.

Library amplification was performed using KAPA HiFi polymerase (Roche Diagnostics Co., Indianapolis, IN, USA) under the following PCR conditions: 95 °C for 3 min; 12 cycles of 98 °C for 20 s, 60 °C for 15 s, and 72 °C for 30 s; followed by a final extension at 72 °C for 10 min, and held at 4 °C. The amplified PCR product was purified using 0.8× bead purification [12,13].

### 2.5. Library Pooling and DNA Nanoball (DNB) Sequencing

DNA library was allocated to 40 million reads. The pooled DNA library was then denatured at 95 °C for 6 min to generate single-stranded DNA, followed by circularization using ligase at 37 °C for 30 min. The resulting single-stranded circularized DNA (circDNA) library was transformed into DNA nanoballs (DNBs) using DNB polymerase I, provided by the DNBSEQ-G50RS FCL SE50 sequencing kit (MGI, Shenzhen, China), at 30 °C for 25 min. Sequencing was performed on the DNBSEQ-G50RS platform using the DNBSEQ-G50RS sequencing flow cell [12,13,14].

### 2.6. Bioinformation Data Analysis and Interpretation

High-throughput sequencing reads underwent rigorous quality filtering prior to downstream analysis. Reads shorter than 35 base pairs, reads with low-quality scores (Phred < Q20), PCR duplicates, and low sequence complexity were removed using Trimmomatic (v0.39) and PRINSEQ (v0.20.4), ensuring that only high-fidelity data were retained for subsequent analysis. To minimize host background, filtered reads were first aligned against the Telomere-to-Telomere human reference genome (T2T-CHM13, v2.0) [15] using the Burrows-Wheeler Aligner (BWA-MEM, v0.7.17) [16] with default parameters (Table S1).

Reads unmapped to the human genome were subsequently extracted and subjected to secondary alignment against a comprehensive microbial reference database using BWA-MEM. The microbial reference database comprised a non-redundant set of 5884 viral genomes, 18,432 bacterial genomes, 3146 fungal genomes, and 370 parasitic genomes. All reference sequences were manually selected and curated from the NCBI GenBank, RefSeq, and Virus databases prioritizing entries with high quality samples and genomes.

Pathogens were considered as potential candidates if they met the following stringent criteria. For most taxa, a normalized abundance (measured as transcripts per million, TPM) at least ten-fold higher than the maximum observed in any matched controls (extraction controls, library preparation controls, or no-template control). Low-yield taxa with known extraction or sequencing challenges, e.g., RNA viruses and obligate intracellular or slow-growing bacteria such as Mycobacterium tuberculosis, a minimum of one uniquely mapping reads per organism and complete absence of reads for the same taxon in all matched controls [17]. To prevent artifacts from repetitive elements, we evaluated the uniformity of read mapping across each reference genome by computing the Shannon entropy of read-placement, where taxa with low entropy (<0.8) indicative of clustered mappings were excluded from further consideration.

### 2.7. Management of Background Contamination

A longitudinal contaminant database was established to catalog potential background organisms and artifacts identified in external positive controls, negative controls, and non-template controls (NTCs). During the bioinformatic analysis, sequence reads aligning with taxa listed in this database were automatically flagged for supervisory review. Final adjudication of organisms detected above positivity thresholds was performed by the laboratory director to differentiate true pathogens from environmental noise or artifacts. This decision-making process relied on three primary criteria: (i) historical presence of the organism in the contaminant database; (ii) simultaneous detection of multiple common environmental species within the sample; and/or (iii) evidence of well-to-well cross-contamination from high-titer samples within the same sequencing run. potential signal bleed-over from high-titer samples processed in the same run. Following this review, any organism identified as a contaminant was disregarded, and the specimen was deemed negative for that specific pathogen in the final analysis.

### 2.8. Statistical Analysis

The statistical analysis in this study employed a comprehensive approach to evaluating the performance of metagenomic next-generation sequencing (mNGS) compared to conventional microbiology tests (CMT) in detecting pathogens in PJI. McNemar’s chi-squared test was used to assess differences in pathogen detection rates, while paired t-tests compared positive detection rates and the number of pathogens detected between the two methods. The Wilcoxon signed-rank test was also used alongside the paired t-test to compare the number of pathogens detected, providing a non-parametric alternative. Additionally, sensitivity and specificity calculations were performed to evaluate the overall performance of mNGS in pathogen detection. These statistical methods collectively provided a thorough assessment of mNGS’s capabilities, particularly in culture-negative scenarios, offering a multi-faceted comparison with conventional microbiology tests for PJI pathogen detection.

## 3. Results

### 3.1. Cohort Characteristics

A total of 44 patients with suspected PJI were screened across seven participating hospitals, with 42 patients meeting the inclusion criteria and enrolled in the study. The distributions of enrolled cases were: Chi-Mei Medical Center (*n* = 10), National Taiwan University Hospital (*n* = 8), Kaohsiung Veterans General Hospital (*n* = 8), Far-Eastern Memorial Hospital (*n* = 6), Taichung Veterans General Hospital (*n* = 6), Shin-Kong Memorial Hospital (*n* = 3), and National Taiwan University Hospital Hsin-Chu Branch (*n* = 1).

The demographic and clinical characteristics of the study cohort (*n* = 42) are summarized in Table 1. The mean age of the patients was 67.9 ± 14.6 years, with an equal distribution of males and females (50% each). The most common presenting clinical manifestation was new-onset pain (69.1%), followed by fever (28.6%) and wound dehiscence (19.1%). Hypertension (66.7%) and diabetes (35.7%) were the most common underlying conditions. The knee was the most common joint (69.1%, combining right and left), followed by the hip (19.0%). Most infections occurred late, with 40.5% presenting more than 24 months after implantation and 26.2% between 3 and 24 months. Notably, a high proportion of patients (78.6%) had received antibiotics within the two weeks prior to sample collection. Baseline laboratory profiles were highly variable, reflecting the heterogeneous clinical spectrum (Table 2).

### 3.2. Number of Pathogen Detection Details and Overlap

**Figure 1:**
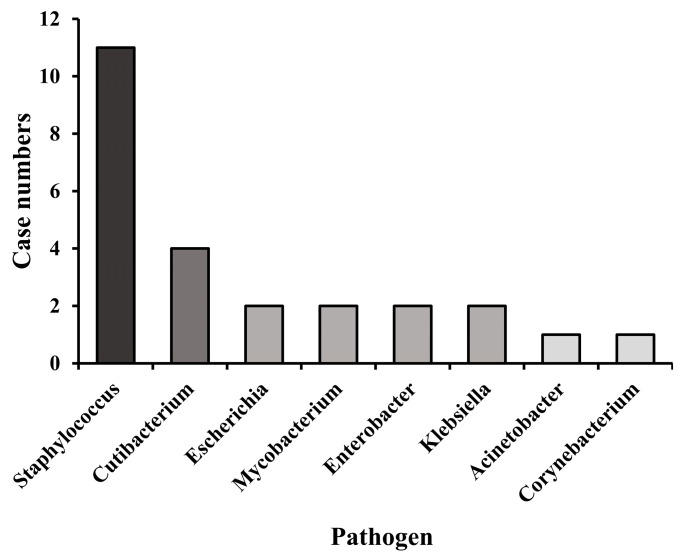
Number of bacterial genera identified by metagenomic next-generation sequencing (mNGS) in the 28 mNGS-positive patients of the cohort.

**Figure 2:**
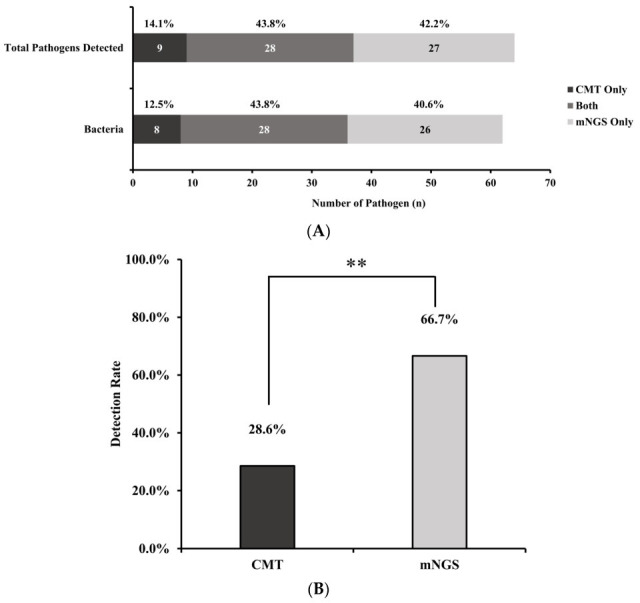
(**A**) Distribution of causative pathogens number between conventional microbiology tests (CMT) and metagenomic next-generation sequencing (NGS). (**B**) Positivity of pathogen number detection between the two methods. Asterisks (**) denote a statistically significant difference with a *p*-value < 0.01 (Paired *t*-test *p* = 0.0046, α = 0.05, Difference = 38.1%).

Conventional wound/joint microbiology-identified organisms, such as *Staphylococcus aureus*, *Pseudomonas aeruginosa*, and *Mycobacterium tuberculosis* complex, were detected in 12 of the 42 enrolled patients. Detailed information is shown in Table 3. The number of pathogens identified by mNGS is shown in Figure 1. *Staphylococcus* was the most common genus detected (*n* = 11 occurrences), followed by *Cutibacterium* (*n* = 4). Other detected genera included *Escherichia*, *Mycobacterium*, *Enterobacter*, *Klebsiella* (*n* = 2 each), *Acinetobacter*, and *Corynebacterium* (*n* = 1 each). The difference in the identified pathogens between the CMT and mNGS results is shown in Figure 2. When considering the total number of detected pathogens (including bacteria and fungi), 42.2% were identified exclusively by mNGS, 14.1% exclusively by CMT, and 43.8% were detected by both methods. A similar pattern was observed when analyzing the number of bacterial pathogens specifically: 40.6% were detected by mNGS only, 12.5% by CMT only, and 43.8% by both techniques.

### 3.3. Diagnostic Performance of mNGS Versus Conventional Microbiology

The overall diagnostic performance for pathogen detection was compared between mNGS and CMT. Before statistical comparison, each paired result was classified into four clinical categories: (a) concordant positive: mNGS matched clinical findings/CMT with no additional pathogens detected; (b) concordant positive extra: mNGS matched clinical findings and detected ≥ 1 extra pathogen not found by CMT; (c) discordant: pathogen detected by CMT but not by mNGS; and (d) concordant negative: neither method detected a pathogen. As shown in Table 4, mNGS demonstrated a significantly higher positive detection rate than CMT. mNGS identified pathogens in 28 of the 42 patients (66.7%), while CMT yielded positive results in 12 of the 42 patients (28.6%). This difference in detection rates was statistically significant (McNemar’s test, *p* = 0.01). Analysis of paired results revealed concordance in 16 cases (7 tested positive by both methods, 9 tested negative by both methods) and discordance in 26 cases. Specifically, mNGS detected pathogens in 21 cases that were negative for CMT, while CMT identified pathogens in 5 negative cases for mNGS.

### 3.4. Performance in Culture Negative Cases and Laboratory Findings

Highlighting the utility of mNGS in challenging diagnostic scenarios, the analysis showed that among the 30 patients with negative CMT results, mNGS successfully identified potential pathogens in 21 (70.0%). General laboratory findings indicated ranges consistent with inflammatory processes in many patients, including elevated high-sensitivity C-reactive protein (hsCRP) and erythrocyte sedimentation rate (ESR), though specific mean or median values are not presented.

### 3.5. Clinical Impact Assessment

To assess the perceived clinical utility of mNGS, treating clinicians surveyed each enrolled patient to determine whether the mNGS results provided additional information relevant to improving medical decisions. As shown in Table 5, a substantial majority of clinicians (30/42, 71.4%) responded affirmatively, indicating that the information obtained from mNGS was beneficial for guiding patient management in this study. Conversely, 28.6% (12/42) did not find that the mNGS results added value to their decision-making process in their cases.

## 4. Discussion

The diagnosis of PJI remains a significant clinical challenge, hampered by the limitations of conventional culture techniques, particularly in the context of culture-negative PJI [9,18]. This prospective, multicenter study evaluated the diagnostic utility of mNGS compared to CMT in a cohort of 42 patients with suspected PJI in Taiwan. Our findings demonstrate that mNGS significantly enhances the rate of pathogen detection compared to CMT, offers substantial diagnostic yield in culture-negative cases, identifies a broader spectrum of microorganisms, and provides information perceived as clinically valuable by treating physicians.

The primary outcome of this study revealed a striking difference in pathogen detection rates. mNGS identified potential pathogens in 66.7% of patients, more than double the 28.6% detection rate achieved by CMT. This substantial increase (38.1% absolute difference) strongly supports our hypothesis that mNGS is superior to conventional culture for pathogen identification in this setting. When considering overall positivity (detection by either method), mNGS achieved a 71.4% positivity rate compared to 40.5% for CMT, further highlighting its enhanced sensitivity. These findings align with a growing body of literature demonstrating the advantages of mNGS in orthopedic and other infections [10,11,19]. While direct comparisons vary, our observed difference appears larger than that reported in a similar PJI cohort [20]. This variation might stem from differences in patient populations, the prevalence of fastidious organisms, local CMT protocols, or the specific mNGS workflow and bioinformatics pipeline used. Notably, the high rate of prior antibiotic exposure in our cohort (78.6%) likely suppressed bacterial growth in culture more profoundly than it affected DNA detection by mNGS, potentially amplifying the observed difference in detection rates [21].

A critical advantage of mNGS highlighted by our results is its ability to diagnose CN-PJI. Conventional cultures were negative in nearly 60% of our cohort, reflecting the common diagnostic dilemma faced by clinicians. Within this substantial group of 25 culture-negative patients, mNGS successfully identified potential pathogens in 64.0%. This ability to provide microbial identification when culture fails is paramount, as it can facilitate the transition from broad-spectrum empirical therapy to targeted antimicrobial treatment, potentially improving efficacy, reducing toxicity, and mitigating the development of resistance [22].

While demonstrating superior detection, the interpretation of mNGS results requires careful consideration, particularly regarding specificity. Our analysis yielded a high sensitivity (82.4%) but a relatively low specificity (36.0%) when using a binary positive/negative framework. This low specificity is a well-recognized challenge associated with highly sensitive molecular methods like mNGS [23]. Several factors likely contribute: the detection of residual DNA from non-viable organisms (especially relevant given the high rate of prior antibiotic use), the identification of microorganisms that are part of the colonizing skin or wound microbiota but not causative agents of the PJI, and potential low-level environmental or reagent contamination during sample processing [24]. Furthermore, discrepancies between CMT and mNGS, such as the absence of certain culture-positive pathogens like *Pseudomonas aeruginosa* or *Prevotella disiens* in mNGS reports, may arise from stringent bioinformatics filtering. If the number of specific microbial reads fails to meet the predefined threshold, the pathogen is not reported to maintain high diagnostic rigor. This underscores the necessity of interpreting mNGS results not in isolation but integrated with the patient’s clinical presentation, imaging findings, inflammatory markers, and CMT results. The moderate negative predictive value (75.0%) suggests that a negative mNGS result provides reasonable confidence in excluding detectable infections, while the modest positive predictive value (46.7%) reinforces the need for clinical correlation for positive findings.

Furthermore, mNGS provided insights into the polymicrobial nature and diverse etiology of PJI. It detected significantly more pathogen instances than CMT and identified a wider array of organisms. While common PJI pathogens like *Staphylococcus aureus* and *S. epidermidis* were frequently detected, consistent with established PJI microbiology (7), mNGS also identified organisms known to be fastidious or difficult to culture, such as *Cutibacterium* species and *Mycobacterium* species, which can be missed by routine CMT. The detection of a virus, although a single instance, also highlights the pan-pathogen potential of mNGS beyond the scope of standard bacterial culture.

Despite the complexities of interpretation, the clinical utility of mNGS was affirmed by the treating physicians in this study. Over 71% indicated that the results provided additional useful information relevant to clinical decision-making. This suggests that even with the recognized specificity challenges, the granular microbial information provided by mNGS is often perceived as actionable, potentially influencing antibiotic selection, therapy duration, or surgical management strategies [25]. The high sensitivity of mNGS enables a rapid shift from broad-spectrum empirical antibiotics to targeted therapy, which is vital for patients with culture-negative results. By identifying fastidious organisms early, clinicians can optimize surgical timing and select the most effective antimicrobial agents, ultimately reducing the risk of treatment failure.

This study has several strengths, including its prospective, multicenter design, which involves diverse hospital settings across Taiwan, enhancing the generalizability of the findings. The use of the standardized 2021 EBJIS/ESCMID criteria for PJI diagnosis provides a robust clinical framework. Samples were processed simultaneously for both mNGS and CMT, allowing for direct comparison. However, limitations must be acknowledged. The sample size is relatively small for a multicenter trial. The extremely high rate of prior antibiotic use (78.6%) complicates direct comparisons with culture and may overestimate the relative advantage of mNGS compared to a cohort with less antibiotic exposure. While standardized criteria have been used for PJI diagnosis, a universally accepted “gold standard,” especially for culture-negative PJI, remains elusive, making definitive performance calculations challenging. We did not systematically analyze the correlation between specific mNGS read counts/metrics and clinical significance, nor did we perform cost-effectiveness or turnaround time comparisons, which are critical for clinical implementation. Finally, the potential for contamination, inherent to sensitive molecular assays, requires ongoing vigilance in laboratory practice and bioinformatics filtering.

In conclusion, this multicenter prospective study provides compelling evidence that mNGS significantly enhances pathogen detection in patients with suspected PJI compared to conventional microbiological culture, particularly in the challenging scenario of culture-negative infections. It identifies a broader range of potential pathogens, including fastidious organisms. While interpretation requires careful integration with clinical data due to specific considerations, the information provided by mNGS is often deemed valuable by clinicians for guiding patient management. mNGS is a powerful adjunctive diagnostic tool that, when used judiciously, can improve the accuracy and timeliness of PJI diagnosis, leading to more targeted therapies and potentially better patient outcomes. Future research should focus on establishing standardized interpretation guidelines, refining bioinformatics pipelines to better distinguish pathogens from contaminants or colonizers, evaluating cost-effectiveness, and defining the optimal role of mNGS within comprehensive PJI diagnostic algorithms.

## Figures and Tables

**Table 1:** The demographic and clinical characteristics of the 42 enrolled patients.

Demographic Characteristics	
Age, mean ± standard deviation (years)	67.9 ± 14.6
Gender, n (%)	
Female	21 (50.0)
Male	21 (50.0)
Clinical Manifestations, n (%)	
Fever	12 (28.6)
New-onset pain or swelling	29 (69.1)
Wound dehiscence	8 (19.1)
Underlying condition, n (%)	
Hypertension	28 (66.7)
Ischemic heart disease	4 (9.5)
Diabetes mellitus	15 (35.7)
Body mass index ≥ 30 kg/m^2^	6 (14.3)
Chronic heart failure	5 (11.9)
Chronic pulmonary disease	3 (7.1)
Peripheral vascular disease	6 (14.3)
Malignancies	6 (14.3)
Received immunosuppression medications	3 (7.1)
Joint site, n (%)	
Right Knee	18 (42.9)
Left Knee	11 (26.2)
Right Hip	3 (7.1)
Left Hip	5 (11.9)
Other	5 (11.9)
Original operation, n (%)	
Knee replacement	26 (61.9)
Hip replacement	9 (21.4)
Other	7 (16.7)
Surgical intervention before sampling, n (%)	20 (47.6)
Post-implantation days, n (%)	
<3 weeks	6 (14.3)
3 weeks–3 months	8 (19.1)
3–24 months	11 (26.2)
>24 months	17 (40.9)
Antibiotics given before sampling within 2 weeks, n (%)	33 (78.6)

**Table 2:** Baseline laboratory profiles of the 42 enrolled patients.

Laboratory Testing, Median (Range)	
Hemoglobin (g/dL)	10.7 (7.3–15.4)
White blood cell (WBC) (μL)	8200 (2720–22,340)
Platelet (×10^3^/μL)	302.0 (97–455)
Creatinine (mg/dL)	0.9 (0.37–21)
Blood urea nitrogen (mg/dL)	16.0 (7.8–130)
High sensitivity C-reactive protein (mg/dL)	9.0 (0.507–111.2)
Glucose (mg/dL)	112.5 (82–286)
Erythrocyte sedimentation rate (mm/h)	64.0 (17–120)
Joint fluid total WBC (μL)	8560 (1210–68,286)

**Table 3:** The number of pathogens identified by positive conventional culture results from 12 patients in the cohort.

Wound Pus/Joint Fluid Cultures	No. of Pathogens (%)
Bacteria	
* Staphylococcus aureus*	4 (9.52)
*Pseudomonas aeruginosa*	3 (7.14)
*Streptococcus agalactiae*	2 (4.76)
*Prevotella disiens*	2 (4.76)
* Staphylococcus capitis*	1 (2.38)
Coagulase neg. staphylococci	1 (2.38)
* Klebsiella pneumoniae*	1 (2.38)
Mycobacterium	
*Mycobacterium tuberculosis* complex	4 (9.52)
*Mycobacterium* species	3 (7.14)
* Mycobacterium abscessus*	1 (2.38)
Fungi	
* Candida parapsilosis*	2 (4.76)

**Table 4:** Analysis of paired results of 42 enrolled patients by McNemar’s Chi-squared test.

N = 42		Conventional Microbiology Tests (CMT)	
		Positive	Negative
Metagenomic Next-Generation Sequencing (mNGS)	Positive	7 ^†^	21 ^‡^
	Negative	5 ^§^	9 ^¶^

McNemar’s Chi-squared *p*-value = 0.01. ^†^ Concordant positive: mNGS matched clinical findings/CMT with no additional pathogens detected. ^‡^ Concordant positive extra: mNGS matched clinical findings and detected ≥1 extra pathogen not found by CMT. ^§^ Pathogen detected by CMT but not by mNGS. ^¶^ Concordant negative: neither method detected a pathogen.

**Table 5:** Clinical assessment by query from physician’s response in each enrolled patient. A chi-squared goodness-of-fit test was performed to assess whether the proportion of patients for whom mNGS provided helpful information differed from an expected equal distribution (50%). The observed proportion (71.4%) was significantly higher than expected (χ^2^ = 7.714, df = 1, *p* < 0.01), suggesting that mNGS contributed valuable information to medical decision-making in a statistically significant majority of enrolled patients.

N = 42	Did mNGS Provide More Information and Related to Improve Medical Decision in This Enrolled Patient?	
Answer	Yes	No
Response	30	12
%	71.4%	28.6%

## Data Availability

The datasets generated and analyzed during the current study are not publicly available due to institutional policy and patient confidentiality, but de-identified data are available from the corresponding author upon reasonable request.

## Supplementary Materials

The following supporting information can be downloaded at: https://www.mdpi.com/article/10.3390/microorganisms14010233/s1, Table S1: Summary of Sequencing Reads.
